# Sex-Specific Risk Factors and Predictors of Major Adverse Cardiac and Cerebrovascular Events in Heart Failure with Preserved Ejection Fraction with SARS-CoV-2 Infection: A Nationwide Analysis

**DOI:** 10.3390/jcm14051469

**Published:** 2025-02-22

**Authors:** Sai Prasanna Lekkala, Adil Sarvar Mohammed, Hafeezuddin Ahmed, Meshal Al-Sulami, Jahangir Khan, Rupak Desai, Paritharsh Ghantasala, Hemindermeet Singh, Syed Sohail Ali, Christopher Bianco

**Affiliations:** 1 Department of Internal Medicine, UCHealth Parkview Medical Center, Pueblo, CO 81003, USA; saiprasanna.lekkala@uchealth.org; 2 Department of Internal Medicine, College of Medicine, Central Michigan University, Saginaw, MI 48859, USA; ghant2p@cmich.edu; 3 Department of Internal Medicine, Corewell Health Beaumont Royal Oak, Royal Oak, MI 48073, USA; hafeezuddin.ahmed@corewellhealth.org; 4 Department of Cardiovascular Medicine, West Virginia University, Morgantown, WV 26506, USA; meshal.alsulami@hsc.wvu.edu (M.A.-S.); christopher.bianco@wvumedicine.org (C.B.); 5 Department of Internal Medicine, Covenant Healthcare, Saginaw, MI 48706, USA; jahangir.khan@chs-mi.com; 6 Independent Researcher, Atlanta, GA 30033, USA; drrupakdesai@gmail.com; 7 Department of Cardiovascular Medicine, Mercy St. Vincent Medical Center, Toledo, OH 43608, USA; hemindermeet_singh@mercy.com (H.S.); ssali@mercy.com (S.S.A.)

**Keywords:** COVID-19/SARS-CoV-2, heart failure with preserved ejection fraction (HFpEF), cardiac and cerebrovascular events, gender-based disparities

## Abstract

**Background:** Heart failure with preserved ejection fraction (HFpEF) is a condition with limited large-scale data on the short- and long-term effects of SARS-CoV-2 infection. This study aimed to evaluate the prevalence of major adverse cardiac and cerebrovascular events (MACCEs) in HFpEF patients hospitalized with SARS-CoV-2 and identify sex-specific risk factors and predictors of MACCEs in this population. **Methods:** This retrospective study analyzed HFpEF patients hospitalized with SARS-CoV-2 from the 2020 National Inpatient Sample (NIS) using ICD-10 codes. Patients hospitalized with HFpEF and SARS-CoV-2 were categorized by age (18–44, 45–64, ≥65 years). Multivariate logistic regression was used to adjust for potential confounders, with the statistical significance set at a two-tailed *p*-value < 0.05. **Results**: Among 109,750 HFpEF patients hospitalized with SARS-CoV-2, 31,960 (29.1%) experienced MACCEs. Males experienced a higher rate of MACCEs than females (31.1% vs. 27.5%, OR: 1.20, 95% CI: 1.12–1.28, *p* < 0.001). Adjusted analysis revealed that elderly patients (≥65 years, OR: 1.47, 95% CI: 1.33–1.62) compared with the 45–64 age group and males (OR: 1.20, 95% CI: 1.12–1.28, *p* < 0.001) had a higher risk of MACCEs. Key predictors included prior coronary artery bypass grafting (CABG; OR: 1.15, 95% CI: 1.02–1.30), cancer (OR: 1.24, 95% CI: 1.08–1.42), and chronic kidney disease (OR: 1.15, 95% CI: 1.08–1.23). Subgroup analysis identified additional sex-specific risk factors. In males, hyperlipidemia, obesity, tobacco use disorder, prior stroke/transient ischemic attack (TIA), prior venous thromboembolism (VTE), alcohol abuse, depression, and valvular disease were significant predictors of MACCEs. In females, hyperlipidemia, tobacco use disorder, prior stroke/TIA, prior VTE, and depression were significant predictors. **Conclusions:** HFpEF patients hospitalized with SARS-CoV-2 have a high risk of MACCEs, with male sex, older age, prior CABG, cancer, and chronic kidney disease as key risk factors. This study provides the first large-scale analysis of sex-specific predictors of MACCEs in HFpEF patients hospitalized with SARS-CoV-2. These findings underscore the need for focused research and clinical gender-based strategies to mitigate cardiovascular risks in this unique and high-risk population.

## 1. Introduction

Heart failure (HF) is a complex clinical syndrome with symptoms and signs that result from any structural or functional impairment of ventricular filling or ejection of blood [1]. Symptomatic HF with LVEF > 50% is defined as “heart failure with preserved ejection fraction” (HFpEF) [2]. HF is one of the rapidly increasing health conditions and a significant healthcare concern due to its high prevalence, mortality, morbidity, and cost of care in the United States. More than 6.2 million adults in the United States were reported to have HF, which contributed to 364,000 deaths in 2018, according to the Centers for Disease Control and Prevention (CDC) [3]. HF is the most common cause of hospitalization in the elderly population, and out of incident hospitalized HF events, 47% had preserved ejection fraction, and the incidence of HFpEF is increasing based on long-term trends [4].

Acute respiratory infections, such as influenza and Severe Acute Respiratory Syndrome Coronavirus 2 (SARS-CoV-2), can precipitate exacerbations of HF. They cause pulmonary inflammation and fluid accumulation, increase the workload on the heart, and worsen symptoms in patients with preexisting HF [5]. SARS-CoV-2 can affect multiple organ systems, including the heart, due to its systemic hyperinflammatory nature [6]. The mechanism behind SARS-CoV-2-related cardiac injury is likely multifactorial, involving direct cytotoxicity, increased myocardial oxygen demand due to the hyperinflammatory state, and a prothrombotic state leading to intravascular thrombosis [5]. Additionally, the shared cardiometabolic risk profile and inflammation have been proposed as the common link between SARS-CoV-2 and HFpEF. Previous research has shown that older age and pre-existing comorbidities, such as hypertension, diabetes, and cardiovascular (CV) disease, are more common in hospitalized patients and are related to a more severe course of the disease and higher fatality rates [7]. However, there are only a few studies on the prevalence and impact of SARS-CoV-2 infection in patients with HFpEF and even fewer on gender-based differences. This highlights the importance of large-scale data to identify major adverse cardiovascular and cerebrovascular events (MACCEs) risk factors and gender-based inequalities in SARS-CoV-2 and HFpEF. Despite the extensive data on cardiovascular comorbidities in SARS-CoV-2 patients, few studies have specifically examined the interplay between HFpEF and SARS-CoV-2, particularly regarding sex-based differences in outcomes. This study addresses this gap by providing a comprehensive analysis of predictors of MACCEs in HFpEF patients hospitalized with SARS-CoV-2, emphasizing novel sex-specific risk factors that can inform targeted management approaches. In this study, we used the largest inpatient database publicly accessible in the United States.

## 2. Methods

### 2.1. Data Source

The study sample was acquired from the 2020 National Inpatient Sample (NIS) database. The NIS is the largest all-payer inpatient database and includes a stratified 20% random sample of all nonfederal hospital admissions throughout the United States. This is a large administrative database funded by the Agency for Healthcare Research and Quality [8] (AHRQ). By including data from a diverse range of inpatient settings, the sample reflects a broad spectrum of hospitalized patients, accounting for variations in geographic location, hospital size, and patient demographics. Although the dataset captures only hospitalized patients, it provides invaluable insights into the population of HFpEF patients with SARS-CoV-2 who require inpatient care.

### 2.2. Study Population

HFpEF patients hospitalized with SARS-CoV-2 infection were extracted from the 2020 NIS database using the *International Classification of Diseases, Tenth Revision, Clinical Modification (ICD-10-CM)* diagnosis codes. Specifically, I50.3 was used to identify heart failure with preserved ejection fraction, and B34.2 was used for unspecified coronavirus infection.

To ensure accurate identification of HFpEF, patients were required to have I50.3 as either a primary or secondary diagnosis in the hospitalization record. The classification of HFpEF in administrative databases is consistent with clinical guidelines as coded by the physicians. Similarly, B34.2 (coronavirus infection, unspecified) was used in the absence of more specific SARS-CoV-2 codes early in the pandemic. Patients with suspected but unconfirmed COVID-19 (e.g., codes for symptoms only) were excluded from the analysis to ensure a definitive cohort. Patients without a primary or secondary diagnosis of HFpEF or SARS-CoV-2 were excluded to maintain the specificity of our cohort. The final study population was categorized into three predefined age groups: 18–44 years, 45–64 years, and ≥65 years to assess age-based differences in outcomes.

The prevalence and predictors of major adverse cardiac and cerebrovascular events (MACCEs) in HFpEF patients hospitalized with SARS-CoV-2 infection were analyzed using this sample. The primary endpoint was the prevalence of MACCEs, defined as a composite of all-cause mortality, acute myocardial infarction, cardiac arrest, or acute ischemic stroke during hospitalization.

### 2.3. Statistical Analysis

We evaluated the multivariate predictors of MACCEs in the overall HFpEF cohort with SARS-CoV-2 infection. Subgroup analysis was performed in the male and female sub-populations to identify any significant differences in the impact of SARS-CoV-2 infection and HFpEF on MACCEs, and the analyses were also stratified based on age. Multivariate logistic regression analysis was performed to identify factors such as age, sex, race, the median household income in the zip code, the primary expected payer, elective versus non-elective admissions, hospital bed size, and the region of the hospital. Comorbidities like hypertension, diabetes, hyperlipidemia, obesity, peripheral vascular disease, tobacco use disorder, prior MI, prior PCI, prior CABG, prior TIA, prior SCA, prior VTE, cancer, CKD, acquired immune deficiency syndrome, alcohol use, drug use, depression, chronic pulmonary disease, hypothyroidism, valvular disease, and autoimmune conditions were adjusted to determine the risk of MACCEs. The variables included in the multivariable model were selected based on a combination of clinical relevance, prior evidence in the literature, and their potential role as confounders in the relationship between SARS-CoV-2 infection and MACCEs in patients with HFpEF. Specifically, we considered variables with established or hypothesized associations with MACCEs or differential impacts on outcomes in similar populations, as well as those that were consistently reported in the dataset. This approach ensured that the model captured key predictors while minimizing bias. The odds ratio (OR), 95% confidence interval (CI), and *p* < 0.05 were used to express the results of the logistic regression. The NIS database does not have patient identification information; thus, we did not need institutional review board permission to perform this study. We utilized IBM SPSS Statistics 25.0 (IBM Corp, Armonk, NY, USA) software for all the statistical analyses.

## 3. Results

### 3.1. Demographic Characteristics

**Figure 1:**
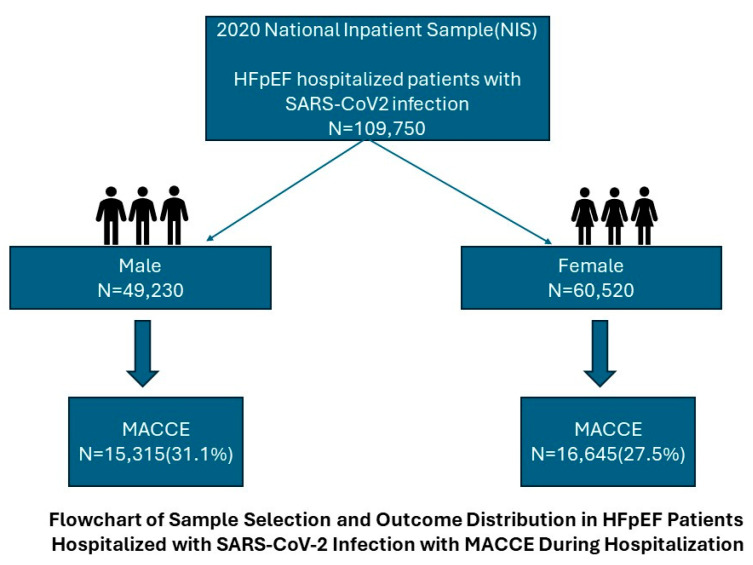
Flowchart of Sample Selection and Outcome Distribution in HFpEF Patients Hospitalized with SARS-Cov-2 Infection with MACCE during Hospitalization.

The study cohort included 109,750 HFpEF patients hospitalized with SARS-CoV-2 infection. Among the sex-specific cohort distribution, a total of 49,230 male patients were identified, of whom 15,315 (31.1%) experienced major adverse cardiac and cerebrovascular events (MACCEs). In contrast, the female cohort included 60,520 patients, with 16,645 (27.5%) experiencing MACCEs (Figure 1). The rate of MACCEs was significantly higher in males compared with females (31.1% vs. 27.5%, odds ratio [OR]: 1.20, 95% confidence interval [CI]: 1.12–1.28, *p* < 0.001). As shown in Table 1 for baseline characteristics, the results highlight significant differences in baseline characteristics and comorbidities between male and female cohorts hospitalized with HFpEF and SARS-CoV-2. The median age of admission was slightly higher in females (median 77 vs. 75 years) compared with males (*p* < 0.001). In terms of racial distribution, Black patients were more represented in the female cohort (23.4%) than the male cohort (18.4%), while White patients were more prevalent among males (66.8%) compared with females (63.1%). Regarding socioeconomic status, as represented by household income quartiles, there was no significant difference between males and females.

In the comparison of comorbidities between male and female cohorts hospitalized with HFpEF and SARS-CoV-2, several key differences were observed. Males exhibited higher prevalence of smoking (32.7% vs. 22.6%), alcohol abuse (2.8% vs. 0.6%), drug abuse (2.3% vs. 1.5%), chronic kidney disease (51.1% vs. 47.0%), and prior myocardial infarction (9.6% vs. 7.6%). Additionally, peripheral vascular disease (9.2% vs. 7.0%) and cancer (6.1% vs. 4.5%) were also more common in males compared with females. Conversely, females showed higher rates of obesity (36.2% vs. 29.9%), depression (17.7% vs. 11.6%), chronic pulmonary disease (42.2% vs. 36.8%), and hypothyroidism (25.7% vs. 12.7%). They were also more likely to have experienced prior venous thromboembolism (8.2% vs. 6.6%) and prior stroke or TIA (10.9% vs. 9.8%). These findings highlight gender-based differences in comorbidity profiles, which may have implications for clinical care and outcomes (Table 1). 

### 3.2. Predictors of MACCEs in the Overall Cohort

Patients aged ≥65 years had a markedly higher adjusted risk of MACCEs (OR: 1.47, 95% CI: 1.33–1.62, *p* < 0.001) compared with the 45–64 age group. Conversely, younger patients aged 18–44 years exhibited a lower adjusted risk (OR: 0.62, 95% CI: 0.47–0.82, *p* < 0.001) compared with those aged 45–64 years. Additionally, male patients were at a higher adjusted risk of experiencing MACCEs than female patients (OR: 1.20, 95% CI: 1.12–1.28, *p* < 0.001). The analysis revealed that specific demographic factors were significantly associated with MACCE risk. Native American (OR: 1.65, 95% CI: 1.15–2.38, *p* < 0.05), Asian/Pacific Islander (OR: 1.37, 95% CI: 1.12–1.67, *p* < 0.05), and Hispanic (OR: 1.27, 95% CI: 1.14–1.42, *p* < 0.001) patients had higher odds of experiencing MACCEs compared with White patients. Similarly, patients in the lowest income quartile were at increased risk of MACCEs compared with those in the highest income quartile (OR: 1.16, 95% CI: 1.05–1.30, *p* = 0.012). Moreover, patients with self-pay as the primary payer showed a significantly higher risk of MACCEs compared with those on Medicare (OR: 1.63, 95% CI: 1.18–2.25, *p* = 0.034). Hospital characteristics also influenced MACCE outcomes. Admissions to large hospitals, as compared with small hospitals, were associated with a higher adjusted risk of MACCEs (OR: 1.18, 95% CI: 1.08–1.29, *p* < 0.001), and urban teaching hospitals had worse outcomes than rural hospitals (OR: 1.33, 95% CI: 1.16–1.53, *p* < 0.001) [Table 1 and Table 2]. 

In this study, males and older age groups were identified as having a higher adjusted risk of MACCEs in HFpEF patients hospitalized with SARS-CoV-2 infection. Additionally, key clinical predictors of MACCEs in the overall cohort included prior coronary artery bypass grafting (CABG; OR: 1.15, 95% CI: 1.02–1.30, *p* = 0.026), cancer (OR: 1.24, 95% CI: 1.08–1.42, *p* = 0.002), and chronic kidney disease (CKD; OR: 1.15, 95% CI: 1.08–1.23, *p* < 0.001). These findings underscore the significant role of pre-existing comorbidities in exacerbating the risk of MACCEs in this population [Table 2]. 

### 3.3. Subgroup Analysis

Sex-specific subgroup analysis revealed distinct predictors of MACCEs among male and female patients. Among males, significant predictors of MACCEs included hyperlipidemia (OR: 1.31, 95% CI: 1.18–1.44, *p* < 0.001), obesity (OR: 1.13, 95% CI: 1.01–1.27, *p* = 0.028), and tobacco use disorder (OR: 1.37, 95% CI: 1.23–1.53, *p* < 0.001). Prior stroke or transient ischemic attack (TIA; OR: 1.30, 95% CI: 1.10–1.53, *p* = 0.002) and prior venous thromboembolism (VTE; OR: 1.47, 95% CI: 1.20–1.80, *p* < 0.001) were also found to be associated with a higher risk of MACCEs in males. Additional predictors included alcohol abuse (OR: 1.53, 95% CI: 1.10–2.12, *p* = 0.001), depression (OR: 1.27, 95% CI: 1.09–1.48, *p* = 0.002), and valvular disease (OR: 1.50, 95% CI: 1.17–1.92, *p* = 0.001).

In the female subgroup, significant predictors of MACCEs included hyperlipidemia (OR: 1.24, 95% CI: 1.14–1.35, *p* < 0.001) and tobacco use disorder (OR: 1.25, 95% CI: 1.13–1.39, *p* < 0.001). Prior stroke or TIA (OR: 1.19, 95% CI: 1.04–1.37, *p* = 0.014) and prior VTE (OR: 1.33, 95% CI: 1.13–1.57, *p* = 0.001) were also associated with increased risk. Additionally, depression was a significant predictor in females (OR: 1.29, 95% CI: 1.14–1.45, *p* < 0.001) [Table 3].

## 4. Discussion

In this retrospective analysis, we assessed risk factors for MACCEs and gender-based disparities in patients with HFpEF and concomitant SARS-CoV-2 infection. To date, most of the existing literature has reported increased comorbidities and cardiovascular diseases in SARS-CoV-2. To our knowledge, the present study is the largest observational study to specifically focus on HFpEF with SARS-CoV-2 along with gender disparities. We identified 31,960 (29.1%) MACCEs in this population.

SARS-CoV-2 is caused by the binding of the surface protein to the human angiotensin-converting enzyme-2 (ACE-2) receptor, which is highly expressed on the surface of pulmonary endothelial cells and cardiomyocytes. Patients with pre-existing HF are particularly susceptible to this infection due to the negative regulatory role of ACE-2 in the activation of the renin–angiotensin–aldosterone system [9] (RAAS). Furthermore, many comorbidities, common in the most severe cases of SARS-CoV-2, are causes of HFpEF [7]. The scoring system used for diagnosing HFpEF included age > 60 years, obesity, atrial fibrillation, pulmonary hypertension, and diabetes. These factors are also contributing to the SARS-CoV-2 severity [10]. This could potentially be the underlying cause of the increased MACCE risk in patients with both HFpEF and SARS-CoV-2.

Our research findings indicate that male gender and older age are the predominant risk factors for MACCEs in hospitalized patients with SARS-CoV-2 and HFpEF. Despite women being 2.8 times more susceptible to HFpEF than men, they exhibit a better survival rate [11]. This difference in outcomes can be attributed to the cardioprotective effects of estrogen in HF, which include inhibiting sympathetic activity and the RAAS [11]. Several factors also contribute to this discrepancy in males with HF, including higher levels of ACE-2 in their bloodstream, which facilitates SARS-CoV-2 entry into cells; increased comorbidities; and weaker defense mechanisms [12]. Consistent with our results, a multicenter study reported that male sex and morbid obesity were significantly associated with higher odds of developing adverse events [12]. Patients over the age of 65 are a more vulnerable population because of the age-related defects in B-cell and T-cell function, adverse effects of polypharmacy, multiple comorbidities, social isolation, and frailty [1,7]. Contrary to our findings, a study by Mansur et al. reported higher mortality rates in men with heart failure with reduced ejection fraction (HFrEF), whereas mortality rates were similar between genders in patients with HFpEF [11]. In contrast, a French observational study found that in-hospital death was more common in male HFpEF patients with COVID-19, with a hazard ratio of 1.58 (1.28–1.95) [9]. These results are consistent with findings from a multicenter study [12] and another study by Narsullah et al. [5]. Our findings correlate with prior literature, which reported increased mortality in the older male population with HFpEF [5,9,12,13]. Our study builds on prior literature by focusing specifically on HFpEF patients with SARS-CoV-2, a subgroup that has been under-represented in previous analyses. By identifying sex-based disparities in MACCE predictors, this study provides actionable insights for tailoring clinical interventions and addressing the unique needs of this population.

A higher risk of developing MACCEs was observed in Native Americans, Asian/Pacific Islanders, Hispanics, and patients in the lower income quartile, which is consistent with prior reported literature [1,14,15]. Among different race groups, Black men had a 1.43-fold higher age-adjusted HF-related cardiovascular disease death rate compared with White men [1]. Additionally, a prospective study by Akwo et al. stated that the patients in the number 1 median household income national quartile were associated with a 12% increase in the risk of HF [1,14]. These differences might be due to a lack of access to or trust in health care and lower socioeconomic status [15]. Therefore, clinicians should be aware of the social and economic determinants of health that may impact the burden of HF.

This study revealed an increased risk of developing MACCEs in HFpEF patients with chronic kidney disease, cancer, and prior CABG. SARS-CoV-2 infection poses a higher risk to the kidneys due to the expression of ACE-2 in proximal tubular cells. This vulnerability can lead to acute kidney injury and exacerbate pre-existing kidney disease [13,16]. A Chinese prospective study by Cheng et al. found that the incidence of death is higher in patients with CKD and SARS-CoV-2 compared with individuals with CKD alone [17]. Patients with a history of cancer usually manifest with muscle wasting, leading to cardiomyopathy, and cancer therapies commonly cause cardiotoxicity [18]. Furthermore, cancer patients’ weakened immune systems make them more vulnerable to SARS-CoV-2 infection and are associated with a higher risk of mortality [18,19]. Additionally, patients with a history of CABG may have other comorbidities and cardiovascular issues, leading to an increased risk of cardiovascular events [20]. The association of CKD, cancer, and CABG with increased MACCE risk highlights the compounding effects of pre-existing comorbidities and SARS-CoV-2 infection in HFpEF patients. These findings underscore the critical need for proactive management strategies to mitigate these risks, particularly in hospitalized HFpEF patients.

As males are at higher risk of developing MACCEs than females, we further determined the predictors associated with MACCEs by performing subgroup analysis based on gender. Our findings align with prior studies with regard to hyperlipidemia, tobacco use disorder, prior stroke, prior venous thromboembolism, and depression being significant predictors among males and females. Males who are current smokers are associated with increased mortality in COVID-19 patients in a meta-analysis of 42 studies that studied the mortality-related risk factors in COVID-19 [21]. Additionally, our study found that smoking is a significant predictor of mortality in both males and females. Meanwhile, obesity, alcohol use, and valvular disease were predictive of MACCEs in the male subset [11,12,21]. These findings underscore the differences in predictors of MACCEs between male and female HFpEF patients hospitalized with SARS-CoV-2 infection. They highlight the importance of individualized approaches to risk assessment and management, tailored to the specific needs and risk profiles of each sex.

Irrespective of comorbidities, HFpEF hospitalized patients with SARS-CoV-2 infection remained at exceptionally high risk of in-hospital mortality compared with those admitted with other respiratory infections like influenza [22,23]. Johnson et al. reported lower hospitalization rates and mortality in vaccinated individuals compared with unvaccinated individuals [24]. Therefore, vaccination against SARS-CoV-2 is recommended by major cardiology societies in Europe and the United States [25,26]. This highlights the importance of future studies to include the role of vaccination in the outcomes of HFpEF with SARS-CoV-2 infection. The results of this study provide critical evidence for sex-specific risk stratification in HFpEF patients with SARS-CoV-2, emphasizing the higher prevalence of MACCEs in males despite the higher baseline prevalence of HFpEF in females. These findings also highlight the need for further research into sex-specific pathophysiological mechanisms and clinical interventions for HFpEF in the context of SARS-CoV-2.

The present study has a few limitations that should be considered. First, potential coding errors in the NIS database may have led to the misclassification of HFpEF and SARS-CoV-2. Additionally, variability in physician documentation and coding practices across different healthcare facilities could influence the accuracy of recorded comorbidities and outcomes. While ICD-10-CM codes are assigned based on physician documentation, they may not fully capture the guideline-based diagnostic criteria of HFpEF (e.g., echocardiographic findings and natriuretic peptide levels). Similarly, to ensure a definitive cohort, patients with suspected but unconfirmed COVID-19 (e.g., those coded only for symptoms without laboratory confirmation) were excluded from the analysis to improve specificity. Second, as it is a retrospective study, it is subject to selection bias. Third, as there is no control group in our study, we could not interpret whether or not SARS-CoV-2 infection was a predictor of adverse events in HFpEF. Fourth, this study lacks long-term follow-up data to assess the impact of long COVID-19 in HFpEF patients, as the NIS database is limited to inpatient data. Long COVID-19, associated with persistent cardiovascular complications, may significantly affect HFpEF patients, warranting future longitudinal studies to explore its long-term effects. Additionally, the applicability of our findings to outpatients may be limited as we included only hospitalized patients. Furthermore, we do not have enough information regarding the vaccination status of the cohorts, which could act as a confounding factor, particularly when considering mortality rates among older and immunocompromised individuals. However, our study’s large sample size enhances its statistical power, which helps mitigate the previously mentioned limitations.

## 5. Conclusions

Our findings conclude that males and the older age group are at higher risk of developing MACCEs in HFpEF patients hospitalized with SARS-CoV-2 infection. This highlights the importance of close monitoring and aggressive treatment of this vulnerable population. When treating these patients, physicians should take into account the risk factors that have been identified, such as cancer, CABG, chronic kidney disease, hyperlipidemia, obesity, tobacco use, stroke or TIA, prior VTE, alcohol misuse, depression, and valvular disease. Addressing these risk factors and sex-based disparities and vaccination can potentially lead to better overall patient outcomes. Further prospective studies are required to explore strategies for reducing in-hospital deaths and improving outcomes. This study offers novel insights by providing the first large-scale analysis of sex-specific predictors of MACCEs in HFpEF patients hospitalized with SARS-CoV-2. These findings underscore the potential to inform sex-specific risk stratification, guide clinical decision making, and improve outcomes in this high-risk population.

## Figures and Tables

**Table 1:** Baseline characteristics of male vs. female cohort hospitalized with HFpEF and SARS-CoV-2, 2020.

Variable		Male (49,230)	Female (60,520)	Total Inpatient Encounters with HFpEF with SARS-CoV-2	*p*-Value
Age (years) at admission, median [IQR]		75 (65–83)	77 (67–85)	76 (67–84)	<0.05
	18–44	2.4%	1.8%	2.1%	
	45–64	20.8%	17.5%	19.0%	
	≥65	76.7%	80.7%	78.9%	
Race	White	66.8%	63.1%	64.8%	<0.05
	Black	18.4%	23.4%	21.1%	
	Hispanic	11.8%	10.7%	11.2%	
	Asian/PI	2.2%	2.1%	2.2%	
	Native American	0.7%	0.7%	0.7%	
Median household income national quartile for patient ZIP code	0–25th	32.2%	33.8%	33.1%	<0.05
	26–50th	28.1%	27.7%	27.9%	
	51–75th	23.8%	22.3%	23.0%	
	76–100th	15.8%	16.2%	16.1%	
Payer type	Medicare	78.7%	81.5%	80.2%	<0.05
	Medicaid	7.2%	7.6%	7.4%	
	Private	12.9%	9.8%	11.2%	
	Self-pay	1.1%	1.0%	1.0%	
	No charge	0.1%	0.1%	0.1%	
Hospital region	Northeast	19.7%	19.4%	19.5%	<0.05
	Midwest	29.7%	28.8%	29.2%	
	South	36.1%	38.8%	37.6%	
	West	14.5%	12.9%	13.6%	
Comorbidities					
Hypertension		88.2%	88.8%	88.6%	0.02
Diabetes		55.5%	53.5%	54.4%	<0.05
Hyperlipidemia		57.0%	54.0%	55.3%	<0.05
Obesity		29.9%	36.2%	33.3%	<0.05
Peripheral vascular disease		9.2%	7.0%	8.0%	<0.05
Smoking		32.7%	22.6%	27.1%	<0.05
Prior MI		9.6%	7.6%	8.5%	<0.05
Prior TIA/stroke		9.8%	10.9%	10.4%	<0.05
Prior VTE		6.6%	8.2%	7.5%	<0.05
Cancer		6.1%	4.5%	5.2%	<0.05
Chronic kidney disease		51.1%	47.0%	48.8%	<0.05
Alcohol abuse		2.8%	0.6%	1.6%	<0.05
Drug abuse		2.3%	1.5%	1.8%	<0.05
Depression		11.6%	17.7%	14.9%	<0.05
Chronic pulmonary disease		36.8%	42.2%	39.7%	<0.05
Hypothyroidism		12.7%	25.7%	19.9%	<0.05
Other thyroid disorders		0.9%	1.5%	1.3%	<0.05
Valvular disease		4.5%	3.4%	3.9%	<0.05
Metastatic cancer		1.4%	0.9%	1.1%	<0.05

HFpEF = heart failure with preserved ejection fraction; SARS-CoV-2 = Severe Acute Respiratory Syndrome Coronavirus 2. IQR = interquartile range, MI = myocardial infarction, TIA = transient ischemic attack, VTE = venous thromboembolism.

**Table 2:** Multivariate predictors of MACCEs in HFpEF patients hospitalized with SARS-CoV-2 infection.

Predictor		Odds Ratio	95% CI		*p*-Value
			Lower	Upper	
Age at admission	18–44 vs. 45–64	0.62	0.47	0.82	<0.001
	≥65 vs. 45–64	1.47	1.33	1.62	
Sex	Male vs. female	1.20	1.12	1.28	<0.001
Race	Black vs. White	1.01	0.92	1.10	
	Hispanic vs. White	1.27	1.14	1.42	<0.001
	Asian/Pacific Islander vs. White	1.37	1.12	1.67	
	Native American vs. White	1.65	1.15	2.38	
Median household income national quartile for patient ZIP code	0–25th vs. 76–100th	1.16	1.05	1.30	0.012
	26–50th vs. 76–100th	1.11	1.00	1.23	
	51–75th vs. 76–100th	1.2	0.92	1.14	
Payer type	Medicaid vs. Medicare	0.93	0.81	1.07	0.034
	Private vs. Medicare	1.02	0.91	1.15	
	Self-pay vs. Medicare	1.63	1.18	2.25	
	No charge vs. Medicare	1.23	0.31	4.92	
Type of admission	Non-elective vs. elective	1.07	0.85	1.34	0.557
Hospital bed size	Medium vs. small	1.13	1.02	1.25	0.001
	Large vs. small	1.18	1.08	1.29	
Hospital location and teaching status	Urban non-teaching vs. rural	1.28	1.10	1.49	0.001
	Urban teaching vs. rural	1.33	1.16	1.53	
Hospital region	Midwest vs. Northeast	0.88	0.79	0.98	0.009
	South vs. Northeast	0.84	0.76	0.93	
	West vs. Northeast	0.93	0.82	1.05	
Hypertension		1.02	0.92	1.13	0.687
Diabetes		0.99	0.92	1.06	0.747
Hyperlipidemia		0.79	0.74	0.84	<0.001
Obesity		0.91	0.85	0.98	0.011
Peripheral vascular disease		0.96	0.85	1.08	0.492
Tobacco use disorder		0.76	0.71	0.82	<0.001
Prior MI		1.06	0.95	1.18	0.301
Prior PCI		0.82	0.55	1.21	0.316
Prior CABG		1.15	1.02	1.30	0.026
Prior TIA		0.81	0.72	0.90	<0.001
Prior SCA		0.86	0.48	1.56	0.627
Prior VTE		0.72	0.63	0.82	<0.001
Cancer		1.24	1.08	1.42	0.002
CKD		1.15	1.08	1.23	<0.001
AIDS		0.61	0.32	1.15	0.124
Alcohol abuse		0.76	0.58	1.01	0.060
Drug abuse		0.96	0.75	1.23	0.746
Depression		0.78	0.71	0.86	<0.001
COPD		0.98	0.92	1.05	0.543
Hypothyroidism		0.96	0.88	1.04	0.272
Valvular heart disease		0.77	0.65	0.91	0.002
Autoimmune conditions		1.00	0.87	1.16	0.963

*p* < 0.05 indicates statistical significance, MI—myocardial infarction, PCI-—percutaneous coronary intervention, CABG—coronary artery bypass grafting, TIA—transient ischemic attack, SCA—sickle cell anemia, VTE—venous thromboembolism, CKD—chronic kidney disease, AIDS—acquired immunodeficiency syndrome, COPD—chronic obstructive pulmonary disease. This multivariate regression analysis included adjustments for demographic factors (age, sex, race, socioeconomic status), healthcare variables (payer type, hospital characteristics, elective status), and an extensive range of health conditions and medical histories, including hypertension, diabetes, hyperlipidemia, obesity, peripheral vascular disease, tobacco use disorder, and a history of various cardiovascular and other medical events (myocardial infarction, percutaneous coronary intervention, coronary artery bypass grafting, transient ischemic attack, sudden cardiac arrest, venous thromboembolism). Furthermore, we considered the presence of comorbidities, including cancer, chronic kidney disease, acquired immune deficiency syndrome, alcohol abuse, drug abuse, depression, chronic pulmonary disease, hypothyroidism, autoimmune conditions, bariatric surgery status, and obstructive sleep apnea.

**Table 3:** Multivariate predictors of MACCEs in subgroup analysis.

MACCE	Odds Ratio	95 Confidence Interval		*p*-Value
		Lower	Upper	
Male subpopulation				
Hyperlipidemia	1.31	1.18	1.44	<0.01
Obesity	1.13	1.01	1.27	0.028
TUD	1.37	1.23	1.53	<0.01
Prior stroke/TIA	1.30	1.10	1.53	0.002
Prior VTE	1.47	1.20	1.80	<0.001
Alcohol abuse	1.53	1.10	2.12	0.011
Depression	1.27	1.09	1.48	0.002
Valvular disease	1.50	1.17	1.92	0.001
Female subpopulation				
Hyperlipidemia	1.24	1.14	1.35	<0.001
TUD	1.25	1.13	1.39	<0.001
Prior stroke/TIA	1.19	1.04	1.37	0.014
Prior VTE	1.33	1.13	1.57	0.001
Depression	1.29	1.14	1.45	<0.001

TUD—tobacco use disorder, TIA—transient ischemic attack, VTE—venous thromboembolism. *p* < 0.05 indicates statistical significance. This multivariate regression analysis included adjustments for demographic factors (age, sex, race, socioeconomic status), healthcare variables (payer type, hospital characteristics, elective status), and an extensive range of health conditions and medical histories, including hypertension, diabetes, hyperlipidemia, obesity, peripheral vascular disease, tobacco use disorder, and a history of various cardiovascular and other medical events (myocardial infarction, percutaneous coronary intervention, coronary artery bypass grafting, transient ischemic attack, sudden cardiac arrest, venous thromboembolism). Furthermore, we considered the presence of comorbidities, including cancer, chronic kidney disease, acquired immune deficiency syndrome, alcohol abuse, drug abuse, depression, chronic pulmonary disease, hypothyroidism, autoimmune conditions, bariatric surgery status, and obstructive sleep apnea.

## Data Availability

The data utilized in this study was obtained from the Healthcare Cost and Utilization Project (HCUP) database, specifically the National Inpatient Sample (NIS) for the year 2020. Access to this dataset is available to researchers through HCUP upon completion of the necessary data use agreements and training. Interested parties can find more information about accessing the NIS dataset at the following link: HCUP Databases (https://hcup-us.ahrq.gov/databases.jsp, accessed on 31 August 2023).
